# Mechanical and Thermal Analysis of Duroplastic Matrix Composites over a Range of Temperatures

**DOI:** 10.3390/polym16050606

**Published:** 2024-02-23

**Authors:** Anna Krzak, Agnieszka J. Nowak, Marcin Heljak, Jerzy Antonowicz, Tushar Garg, Michael Sumption

**Affiliations:** 1 Scientific and Didactic Laboratory of Nanotechnology and Materials Technologies, Silesian University of Technology, 44-100 Gliwice, Poland; agnieszka.j.nowak@polsl.pl; 2 Biomaterials Group, Materials Design Division, Faculty of Materials Science and Engineering, Warsaw University of Technology, 00-637 Warsaw, Poland; marcin.heljak@pw.edu.pl; 3 Semiconductors Division, Faculty of Physic, Warsaw University of Technology, 00-637 Warsaw, Poland; jerzy.antonowicz@pw.edu.pl; 4 Center for Superconducting and Magnetic Materials, Department of Materials Science and Engineering, The Ohio State University, Columbus, OH 43210, USA; garg.206@buckeyemail.osu.edu (T.G.); sumption.3@osu.edu (M.S.)

**Keywords:** laminate, epoxy–glass, polymer, TMA, DMTA, mechanical testing

## Abstract

It is commonly acknowledged that polymer composites in service are often subjected to not only intricate mechanical loads but also harsh environmental conditions. The mechanical and thermal properties of five particular composites are explored here. The composites are composed of laminates of glass cloth type “E” sheet infilled with a duroplastic matrix. This is a thermoset polymer—epoxy resin with different molecular weights. The composites were fabricated by IZOERG company, which is based in Poland. The final articles were 1.5 mm thick by 60 cm long and 30 cm wide, with the glass layers arranged parallel to the thickness. Young’s modulus and tensile strength were measured at room temperature. Using the thermal analysis of dynamic mechanical properties (DMTA), the values of the storage modulus and the loss modulus were determined, and the damping factor was used to determine the glass transition temperature (Tg). It was revealed that the nature of changes in the storage modulus, loss modulus, and damping factor of composite materials depends on the type of epoxy resin used. Thermal expansion is a crucial parameter when choosing a material for application in cryogenic conditions. Thanks to the TMA method, thermal expansion coefficients for composite materials were determined. The results show that the highest value of the coefficient of thermal expansion leads the laminate EP_4_2 based on brominated epoxy resin cured with novolac P. Duroplastic composites were characterized at cryogenic temperatures, and the results are interesting for developing cryogenic applications, including electric motors, generators, magnets, and other devices.

## 1. Introduction

Glass fabric-reinforced composites have been used since the 1970s, and over 30 scientific journal titles are now devoted to these materials. This proves the importance of composite materials in modern industry [1,2]. Glass–epoxy laminates are characterized by several valuable properties, such as high tensile, bending, and compressive strength, good thermal resistance, high melting point, low thermal conductivity, and good electrical insulation. [3,4] It is well known that high stiffness and a low coefficient of thermal expansion make the material particularly attractive for cryogenic applications [3]. In cryogenic conditions, these composites are used as structural elements, fuel tanks, electrical insulation, and load-bearing elements [4,5]. To efficiently develop high-performance epoxy–glass laminates for cryogenic applications, it is necessary to be aware and understand the mechanical and thermal behaviors of composite materials at cryogenic temperatures and how their cryogenic properties are affected by factors such as the content of hardener, filler, and type of matrix [5,6,7]. In the scientific literature [3,4,5,6,7,8,9,10,11,12,13], researchers have repeatedly emphasized the critical role of polymer matrix composite materials, which are widely used in various industries due to their excellent mechanical strength, chemical resistance, and ease of manufacturing complex forms [13]. More advanced composite structures are used in the aerospace industry, exposed to extreme temperatures ranging from −170 °C to 200 °C [14]. Undoubtedly, it is necessary to conduct continuous research and development works to optimize the mechanical properties of composites in cryogenic conditions. Modifying the properties of composite materials by chemical, physical, or physicochemical methods is extremely important from the point of view of modern industry. This is related to the desire to minimize the production costs of an extensive series of products and the possibility of their reprocessing [15,16]. Sapi [3], Schutz [5], and Chen [6] reviewed the properties of composite materials for cryogenic applications. They provided a comprehensive overview of the research development of polymers for cryogenic applications and their thermal, mechanical, and electrical behavior. Elamin [17] analyzed composite sandwich structures’ impact response and damage mechanisms in arctic conditions. This study provides essential background knowledge for the future design of composite sandwich structures with improved low-temperature impact resistance that could be used in Arctic navigation. The Kasen research team [12] carried out mechanical, thermal, and electrical characteristics of cryogenic glass–epoxy laminates named G-10CR and G-11CR. Tests have confirmed that laminates of glass fibers and CR-grade epoxy resin are helpful in construction and insulation applications. Sethi [18] conducted a series of bending tests to investigate the mechanical behavior of glass–epoxy composites at low temperatures (−20 °C, −40 °C, −60 °C, and −80 °C). As a result of his research, he concluded that low temperatures affect interlayer strength. He emphasized that the key to understanding micromechanics and developing physics-based failure criteria is fractographic analysis, which involves the interpretation of fracture morphology to obtain information about material failure.

In the scientific literature, many scientists have attempted the influence of commercially available hardeners on the mechanical properties of epoxy composites for application in cryogenic conditions. Li [19] investigated a newly developed epoxy-functional polysiloxane (PSE) composition to improve the cryogenic mechanical properties of bisphenol F epoxy resin. The results show that adding polysiloxane significantly enhanced the mechanical properties such as tensile strength, impact strength, and fracture toughness. These properties at cryogenic temperature are better than at room temperature. Wu [20] tested three different triethylenetetramine (TETA) cured epoxy resin compositions in different isothermal environments: curing at 80 °C in DSC, curing at room temperature in air, and aging in water at 45 °C. In the Epon 828 (DGEBA)–EPI-CURE 3046 (TETA) system, a higher amount of hardener accelerates cure but results in a looser network structure. The optimal ratio of hardener to resin in a dry environment is a stoichiometric ratio; in a moist environment, it is less than stoichiometric. In his research work, Lv [21] presented data on the thermal conductivity of seven thermosets synthesized from one commercially available dieset (ethylenediglycol diphenyldiglycidyloxide) and seven phenyldiamines to systematically investigate the relationship between thermal conductivity and the molecular structure of the phenyldiamine hardener. “The highest thermal conductivity of 0.27 W/(m K) is obtained from 5-chloro-m-phenyldiamine; the lowest thermal conductivity of 0.14 W/(m K) is obtained with o-phenylenediamine” [21]. Chang [22] developed a method of strengthening and increasing the crack resistance of thermosetting polymers at cryogenic temperatures using CuO (copper oxide) nanotubes. Tensile strength and fracture energy were found to increase with the content of CuO nanotubes up to 4% by weight, improving 18% and 133% at room temperature, respectively, and 21% and 261% at −196 °C, respectively.

The thermo-mechanical analysis is a valuable tool to assess the operational properties of glass–epoxy laminates. Dynamic mechanical analysis (DMTA) allows for a comprehensive characterization of the viscoelastic properties of the tested materials. This method allows the identification of modulus, conservative E′, and loss E′, as well as the phase shift angle δ (tan δ = E′/E′). Knowledge of the variability of the quantities, as mentioned above, as a function of temperature makes it possible to assess the molecular mobility of the tested system [23].

The coefficient of thermal expansion of glass–epoxy laminates is defined as the ratio of the change in the length of the material to the initial length when the temperature changes [23,24,25]. The hardener can increase or decrease the coefficient of thermal expansion depending on the type or amount used. The value of the coefficient of thermal expansion is also affected by the temperature at which the laminate is processed. Hardeners containing large amounts of hydrogen increase the coefficient of thermal expansion of glass–epoxy laminates. In addition, curing agents can affect other thermal properties of glass–epoxy laminates, such as melting point and thermal conductivity [25].

In the scientific literature, you can find several examples that allow you to reduce the thermal expansion of the laminate. One of the simplest ways is the appropriately selected production technology and the use of laminate with less glass content in the structure. The less glass the laminate contains, the lower its thermal expansion [23]. Another way is to use additives such as carbon fibers or glass–ceramic fibers that reduce the thermal expansion of the laminate by increasing its tensile strength [25]. Special chemical additives that affect the thermal properties of the laminate can also be used to reduce its thermal expansion, for example, microfibrillar cellulose [26,27,28].

This research aimed to compare the mechanical and thermal properties of composite materials in different temperature conditions. TMA studies were conducted to understand the thermal expansion of composite materials, which is important in applications where temperature changes are expected—especially in cryogenic conditions. To obtain additional information and characterize the properties of the composite material, DMTA tests were performed to measure the composite materials’ viscoelastic properties. The conducted research is important from the point of view of designing innovative composite materials applied in cryogenic conditions. It is planned to implement composite materials that are to be applicable in conditions of extremely low temperature.

## 2. Experimental Studies

### 2.1. Materials

**Figure 1:**
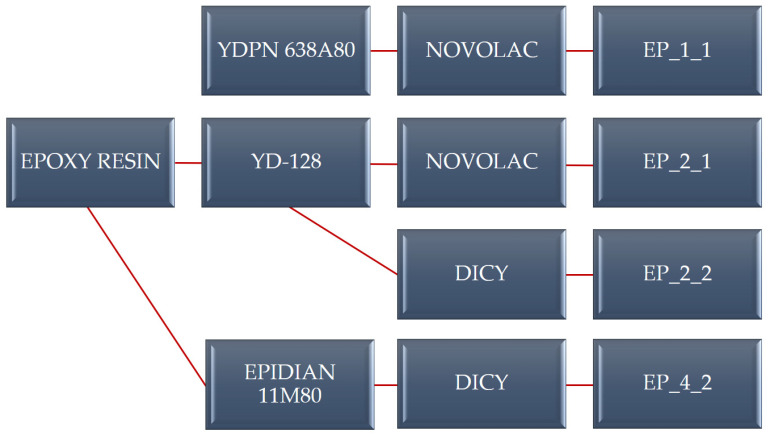
Designation of selected composite materials.

Epoxy resins with the following trade names were used as the matrix for the production of composites: YDPN 638A80 (Kukdo, Seoul, Republic of Korea), YD-128 (Aditya Birla Chemicals, Rayong, Thailand), and EPIDIAN 11M80 (Sarzyna Chemical, Nowa Sarzyna, Poland). Epoxy resins were modified with commonly used hardeners: DICY (Brenntag, Kędzierzyn-Koźle, Poland) and novolac P (Lerg, Pustków, Poland). The reinforcement of the composite materials is the “E” type glass fabric, trade name 7628, made of alumina–borosilicate glass fiber (Joint Stock Company “Steklovolokno”, Polotsk, Belarus) with a weight of 205 g/m^2^. The articles [29,30] provide a detailed composite material production method. The designation of epoxy resins is presented in Figure 1, while Table 1 contains technical data on the glass fabric.

The research considers two commonly available and widely used forms of resin: (1) unmodified resin based on bisphenol A and (2) a solution of epoxy resin enriched with bromine dissolved in an organic solvent.

### 2.2. Preparation

**Figure 2:**
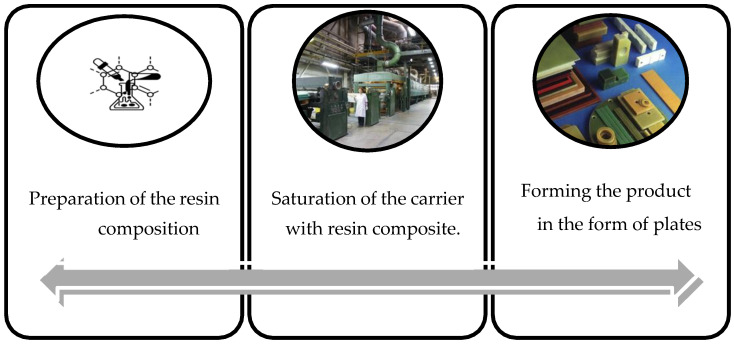
Composite material manufacturing scheme.

**Figure 3:**
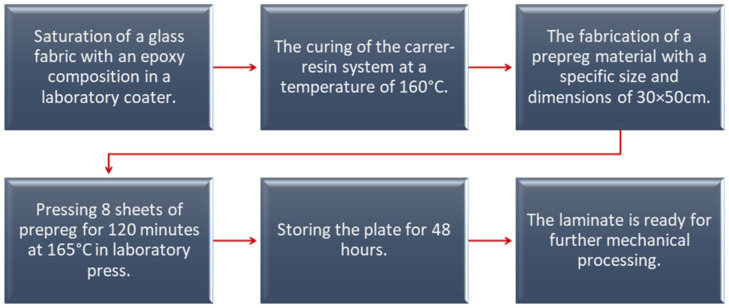
Composite material manufacturing process.

Composite sheets were manufactured at the Plastics Plant IZO-ERG S.A. in Gliwice; the process consisted of the following stages (Figure 2):

The selected components of the matrix composition—resin and hardener—were dissolved in acetone in appropriate proportions. The ingredients were subjected to a mixing process until DICY was completely dissolved. Table 2 shows the parameters of the prepared material—warp. Figure 3 shows the technological process of producing the tested composite material.

Samples were cut out from the prepared composite panels on a guillotine according to standardized sizes.

## 3. Research Methodology

### 3.1. Mechanical Testing

Tests were performed on a universal testing machine (MTS Criterion Model 43) with a 30 kN load cell and mechanical grips. Axial strains were measured employing a laser extensometer (Electronic Instrument Research) with a 50 mm base placed on the middle section of the sample’s gauge length. In contrast, transverse strains (when applicable) were measured using general-purpose strain gauges (EA-06-015EH-120/LE from Micro-Measurements). The tests were conducted in accordance with ASTM D3039 standards [31]. Due to practical limitations, only five specimens for each test were used to obtain the presented results.

### 3.2. DMTA

The subjects of the study were cuboidal samples with dimensions enabling the DMTA test to be carried out in the bending mode with the sample attached at both ends (Dual Cantilever)—60 × 10. During the test, the samples were subjected to a sinusoidal displacement excitation with a constant amplitude of 15 μm in the frequency range from 1 Hz to 20 Hz at a heating rate of 5 °C/min. Based on the data obtained during the test, the modulus dependencies characteristic for the tested samples were determined—conservative E′, loss E″, and the phase shift angle δ on the excitation frequency. Each test was carried out in the temperature range from 20 to 300 °C. The tests were performed by ISO 6721-1:2019-07 “Plastics—Determination of dynamic mechanical properties—Part 1: General principles” [32].

### 3.3. TMA

The coefficient of linear thermal expansion was measured using the TMA Q400 thermomechanical analyzer by TA Instruments. The tests were carried out in the temperature range from −150 °C to 20 °C in the presence of liquid nitrogen; the heating rate of the samples was 10 °C/min. The samples were tested in the warp direction. The tests followed the ISO 11359-2:2021 standard “Thermomechanical analysis (TMA)—Part 2: Determination of coefficient of linear thermal expansion and glass transition temperature” [33].

Based on the following formula [33], the coefficient of thermal expansion was calculated:

(1) $$a=\frac{{x-x}_{0}}{x_{0}\Delta T}=\frac{\Delta x}{x_{0}\Delta T}$$

where

*x*—sample length after temperature change,

*x*_0_—initial length,

*a*—coefficient of thermal expansion,

Δ*T*—temperature increase.

To ensure that the calculations are correct, a regression model was used, and the results obtained were compared.

## 4. Results

### 4.1. Tensile Test

Table 3 lists the tensile strength and Young’s modulus of different samples measured at room temperature.

At room temperature, the composite EP_4_2, which utilizes epoxy–bromine resin + DICY, exhibited the highest values of tensile strength, measuring 583.67 MPa. Conversely, the lowest tensile strength was observed in EP_1_1, consisting of epoxy–bromine resin and novolac. This represents a decrease of 24% compared to EP_4_2. The highest and lowest Young’s modulus values were recorded for EP_2_2 and EP_1_1, respectively, with 25.88 GPa and 22.52 GPa values due to a break in the “extra laminate surface” area, which is considered abnormal.

Comparing the data obtained in this article with the literature data, it can be concluded that promising results have been achieved. For example, the research group Perez [34] studied the tensile properties of 1AIUW001-005 composite and obtained tensile strength and Young’s modulus of 355.8 MPa and 17.51 GPa, respectively. In another study, Kumarasamy [35] investigated the tensile strength of glass fiber-reinforced polymer (GFRP) composites at high and low temperatures. The GFRP laminates were produced using smooth E-glass fabric (800 g/m^2^) and epoxy resin (bisphenol-A). The tensile strength and Young’s modulus were measured at room temperature as 275 MPa and 8 GPa, respectively. On the other hand, Anjaneyulu [36] fabricated composites using epoxy resin with various orientations of glass fibers and presented studies on the effect of symmetric and non-symmetric fiber orientations on the tensile and flexural properties of epoxy laminates reinforced with unidirectional E-glass fibers. The T-2-S-1 laminate with an orientation of 0°/90°/0°/90° exhibited a tensile strength of 211.73 MPa and Young’s modulus of 20.53 GPa. In order to demonstrate examples of damages accross the material, selected images are presented in Supplementary Materials.

### 4.2. Thermal Analysis of Dynamic Mechanical Properties (DMTA)

The glass transition temperature is based on the chemical and molecular structures of the polymer depending on the type and amount of hardener used in the composite. The analysis of the recorded values of the storage modulus and the mechanical loss coefficient indicates differences for the tested materials, distinguished by a different type of epoxy resin and the hardener used.

The graphs presented in Figure 6a–c show the results of the DMTA analysis. On the curves of elastic modulus (E′), loss modulus (E″), and tan δ (tgδ), transformation characteristics of polymeric materials were observed. Table 4 presents the DMTA results for all composite materials.

Figure 6a shows the changes in the conservative storage modulus (E′), loss modulus (E″), and the tan δ (tgδ) for EP_1_1 (black). The storage modulus strongly depends on temperature and decreases as the temperature increases. For EP_1_1, the maximum value of the storage modulus E′ = 15,822 MPa at 99 °C and the loss factors tgδ 0.15 at the glass transition temperature of 122 °C were recorded. The value of the loss modulus is 1521 MPa, but after exceeding 116 °C, it drops significantly.

The following graph (Figure 6a–c) shows the results obtained for the EP_2_1 (red) composite. The value of the storage modulus decreases with increasing temperature, with the most significant decrease taking place after exceeding the temperature of 98 °C, which is 18,035 MPa. The loss modulus value is 2481 MPa, but after exceeding 109 °C, it decreases significantly. The tan δ increases steadily from the start of the test to a temperature of 114 °C. In the further range, there is a significant decrease in this parameter.

The graph (Figure 6a–c) presented for the EP_2_2 (blue) composite containing the DICY hardener maintains a similar shape to those discussed earlier. The value of the storage modulus also decreases with increasing temperature, with the most significant decrease occurring after exceeding the temperature of 135 °C and at the beginning of the transition to the glass phase. The loss modulus (E″) maintains a value of about 1137 MPa, while the highest value of the loss angle occurs at a temperature of 160 °C.

The last DMA graph (Figure 6a–c) shows the results obtained when testing sample EP_4_2 (green). Also, in this case, the course of the charts does not deviate from the predictions. The storage modulus decreases simultaneously with the increase in temperature. The largest decrease is observable after exceeding the temperature of 143 °C. The highest value of the loss angle tgδ was obtained at the temperature of 155 °C.

The presented results of the changes in the storage modulus E′ and the tangent of the loss angle tgδ of various materials based on the duroplastic matrix illustrate the different behavior of materials depending on the shape of the epoxy resin used.

The ability of composite materials to transfer loads is assessed based on the conservative storage modulus [37]. From the graphs, it was noted that as the temperature increases from 20 °C to 250 °C, these curves follow three regions: glassy, transitional, and rubbery. After analyzing these data, a sudden change in the conservative storage modulus in the glass transition region was observed for the following:

- EP_1_1_: 80–160 °C
- EP_2_2_: 100–180 °C
- EP_2_1_: 80–140 °C
- EP_4_2_: 120–190 °C

**Figure 4:**
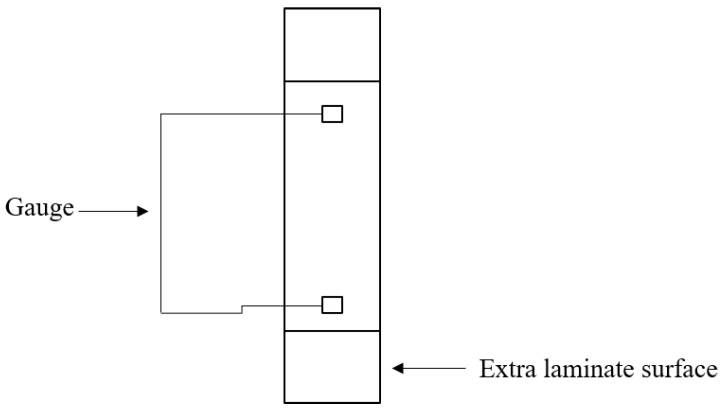
The exemplary form of the sample.

**Figure 5:**
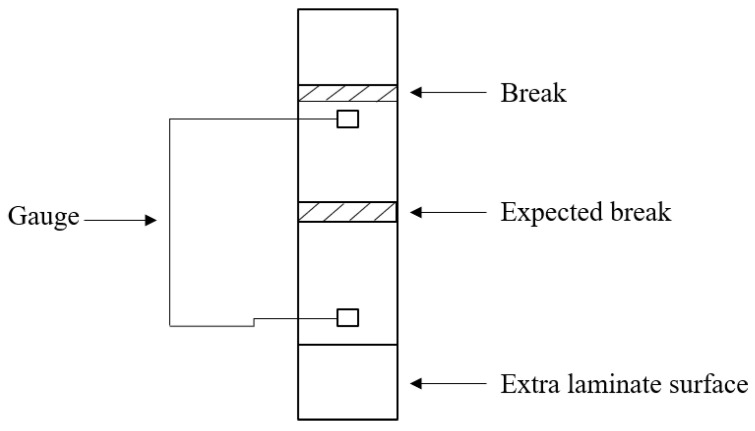
Example of sample break during the test.

**Figure 6:**
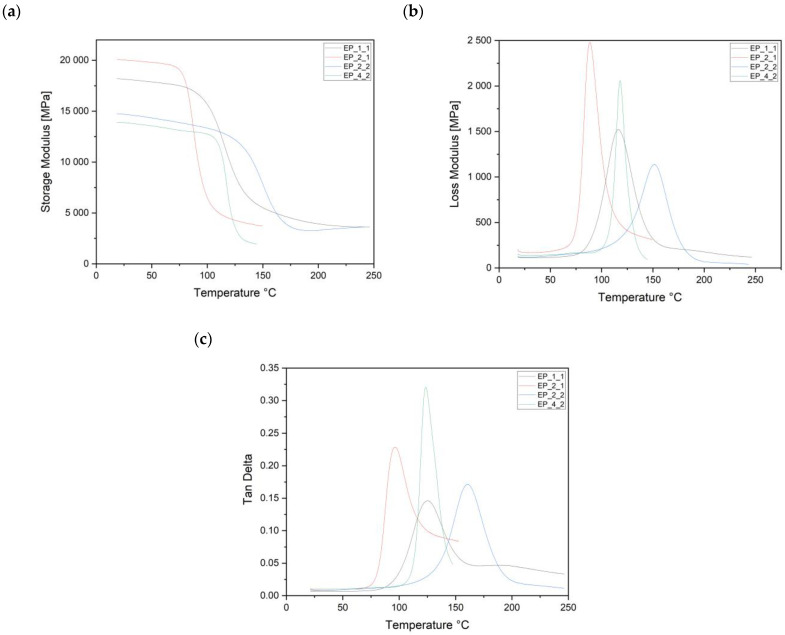
Temperature dependence of storage modulus (**a**), loss modulus (**b**), and tan delta (tgδ) (**c**) for EP_1_1 (black), EP_2_1 (red), EP_2_2 (blue), and EP_4_2 (green).

**Figure 7:**
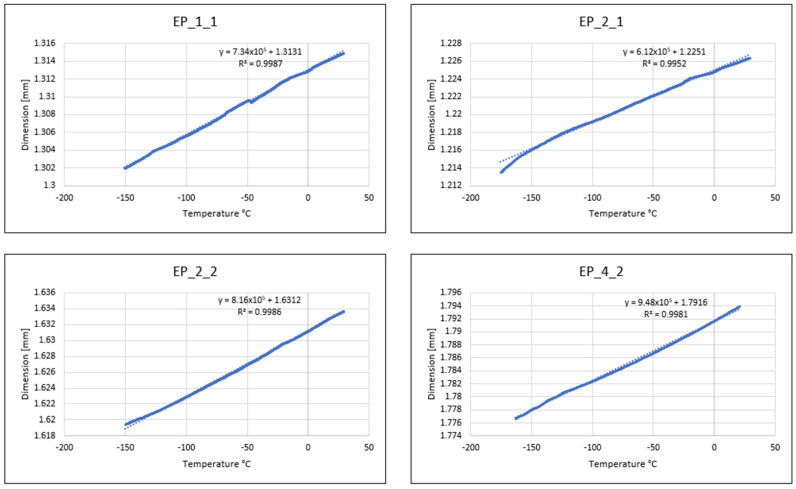
Relative change in length as a function of temperature.

Those changes are most likely caused by hardeners, which change the material’s behavior from elastic to viscoelastic. This is due to the increased number of molecular chains of the duroplastic matrix at high temperatures [25]. At this stage, the main chains in the polymer began to break down and caused significant mechanical losses. For comparison, Shasha Li et al. [26] obtained the optimal value of the glass transition temperature for the composite based on epoxy–novolac resin equal to 75–140 °C. On the other hand, the glass transition temperature of the modified epoxy resin GO–novolac composite ranged from 130 °C to 200 °C [26]. It can be concluded that EP_1_1_ and EP_2_1_, in which the base epoxy resin cured with P novolac was found, fall within the criterion presented by Shasha Li. These figures show that as the temperature increases, the loss modulus rises to Tg, at which E′ then drops dramatically. A further increase in temperature leads to a decrease in the storage modulus due to a reduction in viscosity [38]. The mechanical properties of the composite with duroplastic matrix and reinforced glass fabric depend on the fabric–matrix interfacial bond. The higher the conservative storage modulus and the loss modulus, the better the interfacial adhesion [37]. This behavior is characteristic of cross-linked polymers [25]. The effect of temperature on the phase shift angle (tgδ) at a 1–20 Hz frequency is shown in Figure 4, Figure 5, Figure 6 and Figure 7. These data indicate that Tg of the phase shift angle E′ and E′ occur at different temperatures. The temperature at which the phase shift angle (tgδ) occurs is the highest for all materials. Based on the graph, we can see that the temperature varies from 20°C to 250 °C. Tgδ increases to Tg where there is maximum damping; further temperature increase leads to a decrease outside the tgδ curve. After analyzing the data, a sudden change to the phase shift angle (tgδ) in the glass transition was observed for the following:

- EP_1_1_: 80–180 °C
- EP_2_2_: 100–210 °C
- EP_2_1_: 80–160 °C
- EP_4_2_: 120–190 °C

This can be attributed to the free molecular movement of the polymer chains in the rubber region. The intensity of tgδ max is mainly affected by the reinforcement content (Vf) and orientation [26]. This is due to the polymer chain’s limitations due to the fabric’s presence, which leads to a decrease in tgδ max and a greater interfacial adhesion for the composite material [26].

The tests show that the EP_2_1 composite material is characterized by the highest values of the storage modulus (E′) and the mechanical loss factor (E). The material consists of an unmodified, low molecular weight epoxy based on bisphenol A and novolac P. The graphs show that a phase transition occurs around 100 °C, and this region corresponds to the transformation of the polymer from a glassy state to a viscoelastic state. This transformation is referred to as the glass transition. The polymer becomes flexible in the glass transition region, although it retains some hardness and dimensional stability. The weakest recorded results of storage modulus (E′) and mechanical loss factor (E″) were marked for EP_4_2. It is a composition consisting of a solution of brominated epoxy resin dissolved in methyl ethyl ketone and DICY. A high storage modulus and a low loss modulus indicate that we are dealing with stable and mechanically strong composite materials. The energy that is stored in the material disperses suddenly in the glass transition region, leading to a sharp increase in the loss modulus and a rapid decrease in the storage modulus.

The scientific literature has confirmed that DICY is a stoichiometric hardener for epoxy resins [21]. Composite materials with DICY had the lowest storage modulus. In [22], the authors explain that, at 22 °C, the rate of reaction between the secondary amine and the epoxide is only a quarter of the reaction rate between the primary amine and the epoxide. The initial stage of curing at room temperature is the reaction between the amines and the epoxy, located at the hardener molecule’s two ends. When an excess hardener is used, some of the hardener molecules do not function as bridges between the epoxy molecules, acting as lubricants. This leads to a lower lattice density and, hence, a lower modulus and Tg.

### 4.3. Thermomechanical Analysis (TMA)

Table 5 presents the results of thermal expansion tests of the tested composites, which are graphically summarized in Figure 7.

Based on the results from Table 5, it follows that composite materials EP_1_1_ and EP_4_2_ are characterized by the highest coefficient of thermal expansion: 5.5153 and 5.5127 [10^−5^/°C], respectively. A high coefficient of thermal expansion is unfavorable in developing innovative composite materials. A high coefficient of thermal expansion can also lead to thermal stresses—that is, materials will be exposed to numerous cracks, especially when they are exposed to significant changes in temperature or load.

A low coefficient of thermal expansion was noted in EP_2_1_ and EP_2_2—it amounts to 4.7118 and 4.8752 [10^−5^/°C]. Compared to EP_1_1 and EP_4_2, it is 1.2 times smaller. The influence of DICY and novolac P on the coefficient of thermal expansion of epoxy glass laminates depends on the chemical composition of the epoxy matrix, the method of its preparation, and the proportion of hardeners used. Table 6 below presents the required criteria for the coefficient of thermal expansion of glass–epoxy laminates.

Comparing the test results obtained in this article with the materials of MIL 24768/2, TSE, and G-10, it can be concluded that EP_X_Y is 10 times higher than the values provided by the manufacturers [37,39,40]. This situation is unfavorable as it can lead to the development of multiple cracks in the materials, particularly when they are subjected to significant variations in temperature or load conditions.

## 5. Conclusions

The tests of the developed composite materials were aimed at characterizing the properties, assessing the behavior in the conditions of use provided for them, and determining the temperature dependence of the storage modulus, loss modulus, and mechanical loss tangent. In addition, a study was carried out to determine the coefficient of thermal expansion of composites reinforced with glass fabric. These studies were treated as an introduction to developing new material solutions for cryogenic applications. Based on the conducted research, the following observations were formulated:

- The highest storage modulus values were obtained for the material using novolac-cured type A bisphenol resin. In contrast, the lowest storage modulus values were obtained for the material with brominated epoxy resin cured with dicyandiamide.
- The storage modulus of composite materials, which consist of two different types of epoxy resin (bisphenol A and a product of the reaction between epichlorohydrin and phenol–formaldehyde novolac in methyl ethyl ketone) and are cured with novolac, meets the conventional criterion,
- The lowest coefficient of thermal expansion was recorded for composite materials where epoxy resin based on bisphenol A is used but cured with different catalysts, dicyandiamide, and novolac. The coefficients of thermal expansion are 4.7118 and 4.8752 [10^−5^/°C], respectively.
- Laminates with different types of epoxy resin (phenol–formaldehyde novolac in methyl ethyl ketone and brominated epoxy resin) and different catalysts (novolac and dicyandiamide) are characterized by too high and unfavorable coefficients of thermal expansion.
- The preliminary research presented in this article will allow for the application of several modifications. Additives to reduce thermal expansion should be used for laminates with YDPN 638A80 resin, novolac hardener, YD-128 resin, and DICY hardener.

The next stage of the research will be verifying the developed compositions concerning long-term exposure to liquid nitrogen. Surface roughness testing should also be considered to analyze the morphology and structure of composite materials. Also, it is considered to conduct cryogenic characteristics of composite materials with different reinforcing phases—it is planned to use roving, carbon fiber, and glass–basalt fiber. This will allow for a complete and accurate comparison of materials and selection of the best and economically optimal multilayer composite material that will be used in cryogenic conditions.

## Figures and Tables

**Table 1:** Technical parameters of Joint Stock Company 7628 Type E Glass Fabric.

Parameter	Value
Grammage	205 g/m^2^
Weave type	Plain weave
Glass type	“E”

**Table 2:** Parameters of composite materials.

Symbol	Viscosity [s]	Gel Time [min]	Fluidity [%]	Resin Content [%]
EP_1_1	44.00	2.53	24.60	38.00
EP_2_2	22.00	6.07	13.00	33.60
EP_2_1	118.00	3.44	17.90	35.48
EP_4_2	37.00	9.00	21.00	34.00

**Table 3:** Results of tensile test.

Material	Tensile Strength [MPa]	Young Modulus [GPa]
EP_1_1	443.45	22.52
EP_2_1	544.15	25.48
EP_2_2	551.82	25.88
EP_4_2	583.67	22.84

**Table 4:** DMTA results for four composite material combinations.

Material	Storage Modulus E′ [MPa]	Storage Modulus E′ Peak Maximum [°C]	Loss Modulus E″ [MPa]	Loss Modulus E″ Peak Maximum [°C]	Tan δ [°C]	Tan δ
EP_1_1	15,822	99	1521	116	122	0.15
EP_2_2	11,387	135	1137	154	160	0.25
EP_2_1	18,035	98	2481	109	114	0.20
EP_4_2	11,344	143	2059	151	155	0.32

**Table 5:** Thermal expansion coefficients of the tested composite materials.

Symbol	Linear Expansion Coefficient α in the Temperature Range −150 °C—RT [10^−5^/°C]	Linear Expansion Coefficient α in the Temperature Range −150 °C—RT [10^−5^/°C] Calculated from the Regression Model
EP_1_1_	5.5153 × 10^−5^	5.5890 × 10^−5^
EP_2_1_	4.7118 × 10^−5^	4.6776 × 10^−5^
EP_2_2_	4.8752 × 10^−5^	4.9996 × 10^−5^
EP_4_2_	5.5127 × 10^−5^	5.3334 × 10^−5^

**Table 6:** Parameters of thermal expansion coefficient of glass–epoxy laminates.

Symbol	Linear Expansion Coefficient α [10^−6^/°C]	Company	Reference
MIL 24768/2	0.9	AMERICAN MATERIAL SUPPLY	[37]
TSE	1.0	IZOERG	[39]
G-10	0.83	LAMINATED PLASTICS	[40]

## Data Availability

Data are contained within the article and Supplementary Materials.

## Supplementary Materials

The following supporting information can be downloaded at: https://www.mdpi.com/article/10.3390/polym16050606/s1.
