# Supplementary material for: Mechanical and Thermal Analysis of Duroplastic Matrix Composites over a Range of Temperatures
Source: Polymers (Basel). 2024 Feb 23;16(5):606. doi: 10.3390/polym16050606 (PMC10933775; doi:10.3390/polym16050606)
Supplement: Supplementary file 1 [file polymers-16-00606-s001.zip › polymers-2852232-supplementary.pdf]

Supplementary Materials

In order to demonstrate examples of material structure, selected images have been included in the supplementary document for reference. The images were taken at The Ohio State University with a Olympus GX53 optical microscope, which allows precise analysis of 2D and 3D images at 50x magnification. Tensile test results on damaged specimen show no signs of structural delamination, round dents or air bubbles. The absence of defects indicates proper impregnation of the reinforcing phase with resin and a well-executed manufacturing process with appropriately selected process parameters. In the case of the EP_1_1 sample, it can be observed that the epoxy resin along with the hardener were not completely dissolved and were not thoroughly saturated into the glass fabric.

**EP_1_1**	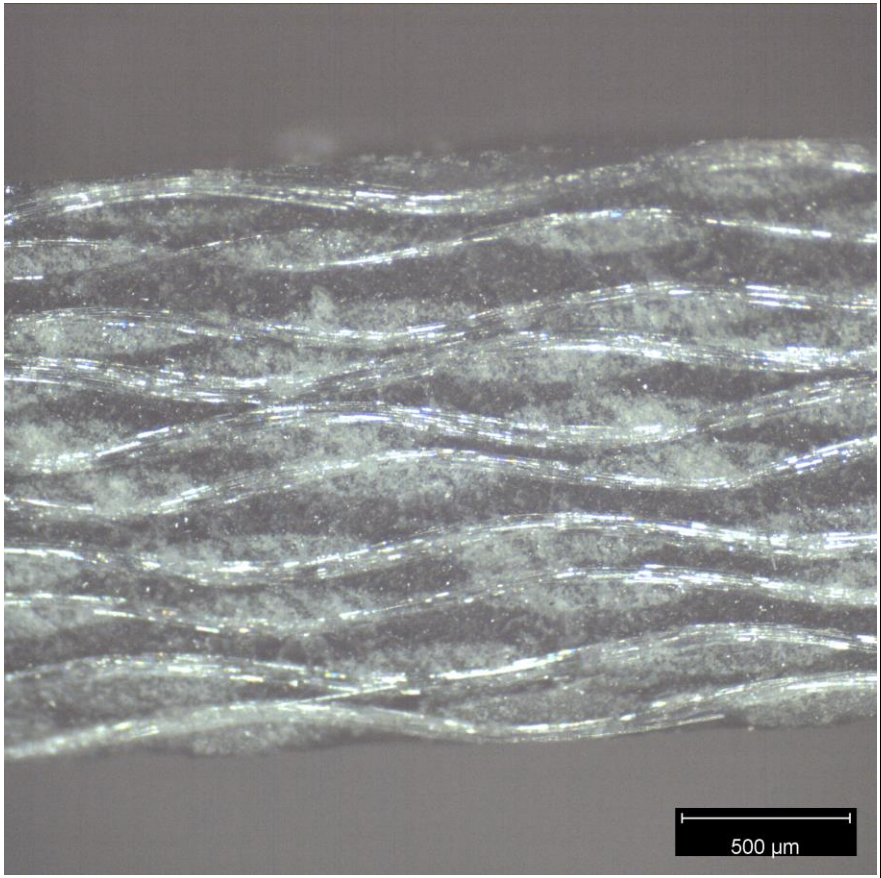	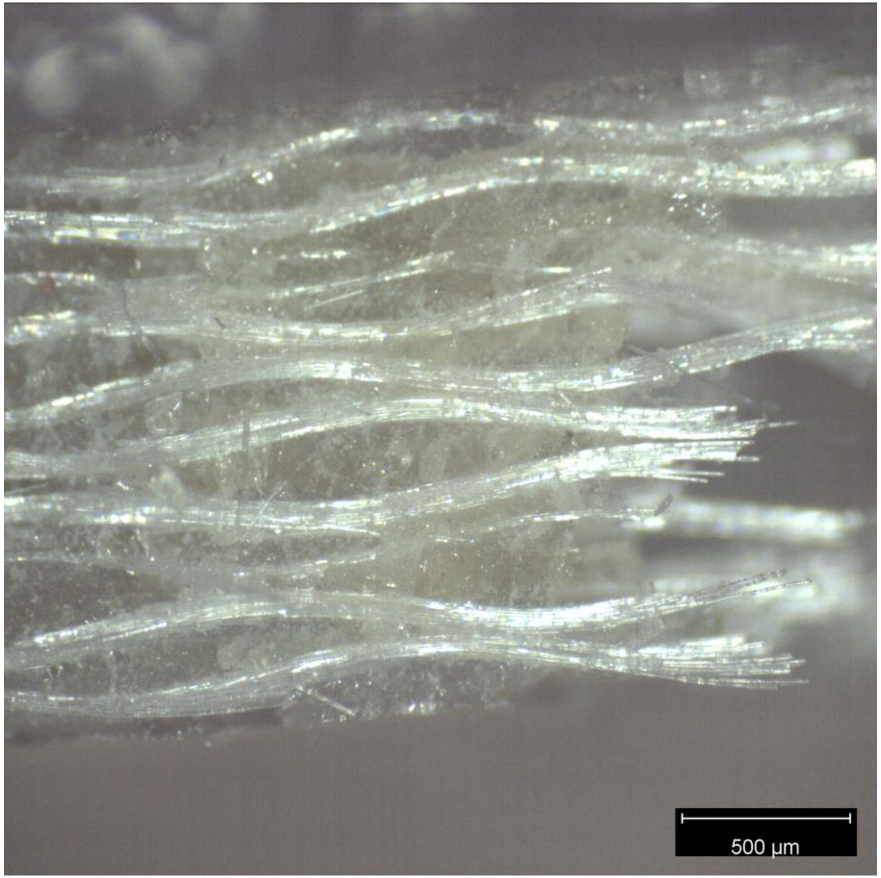

**EP_1_1**	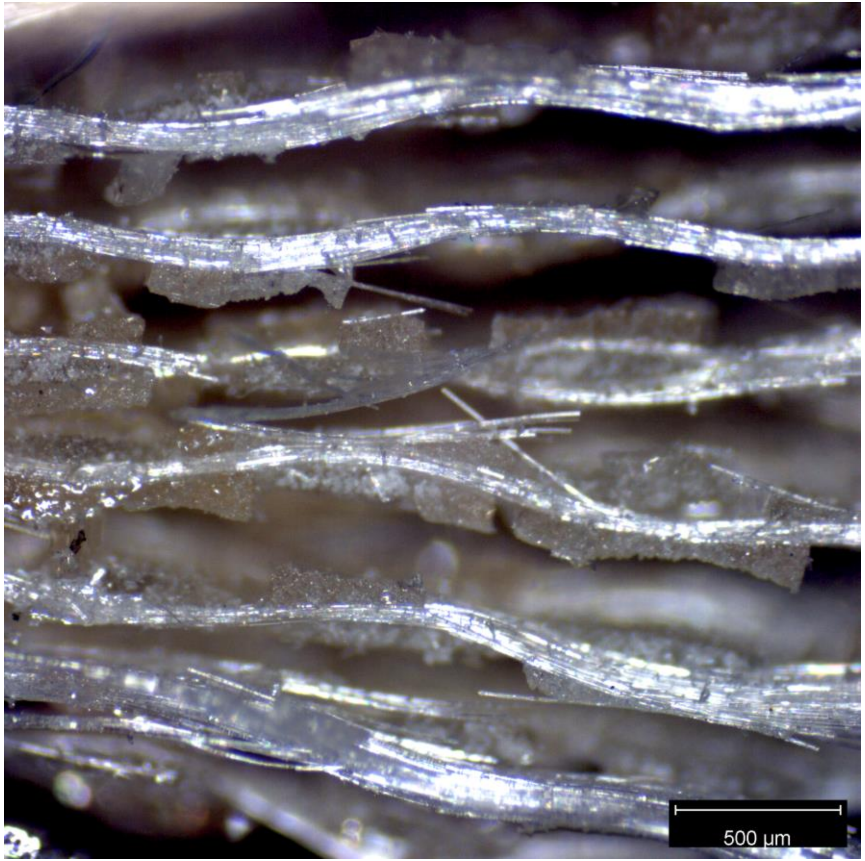	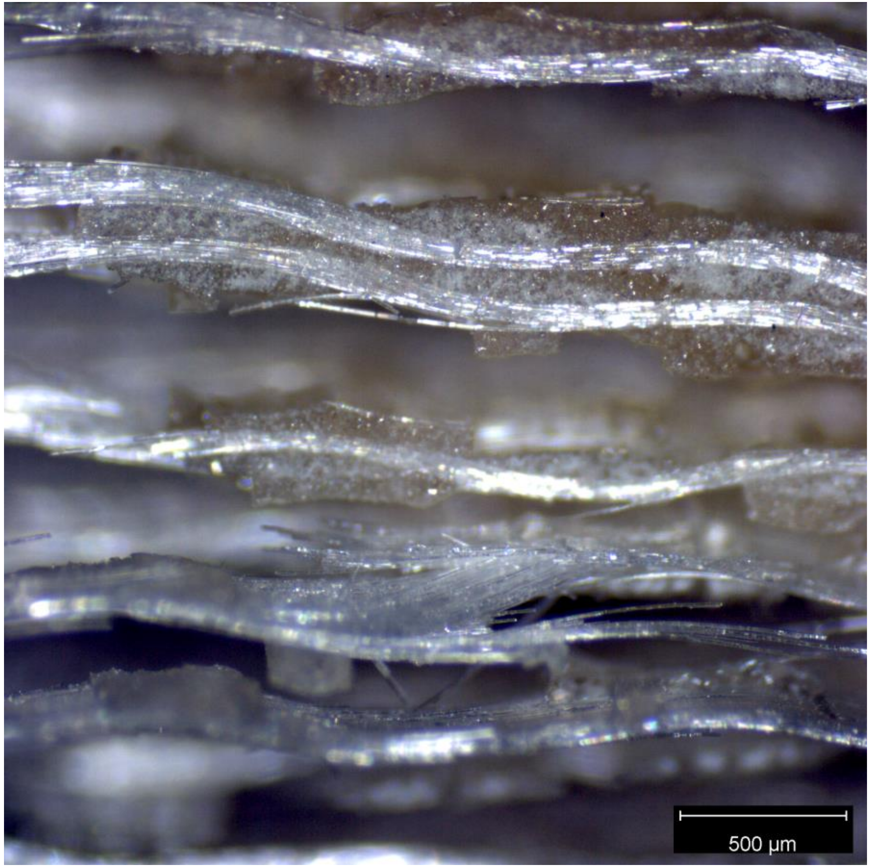

**EP_2_1**	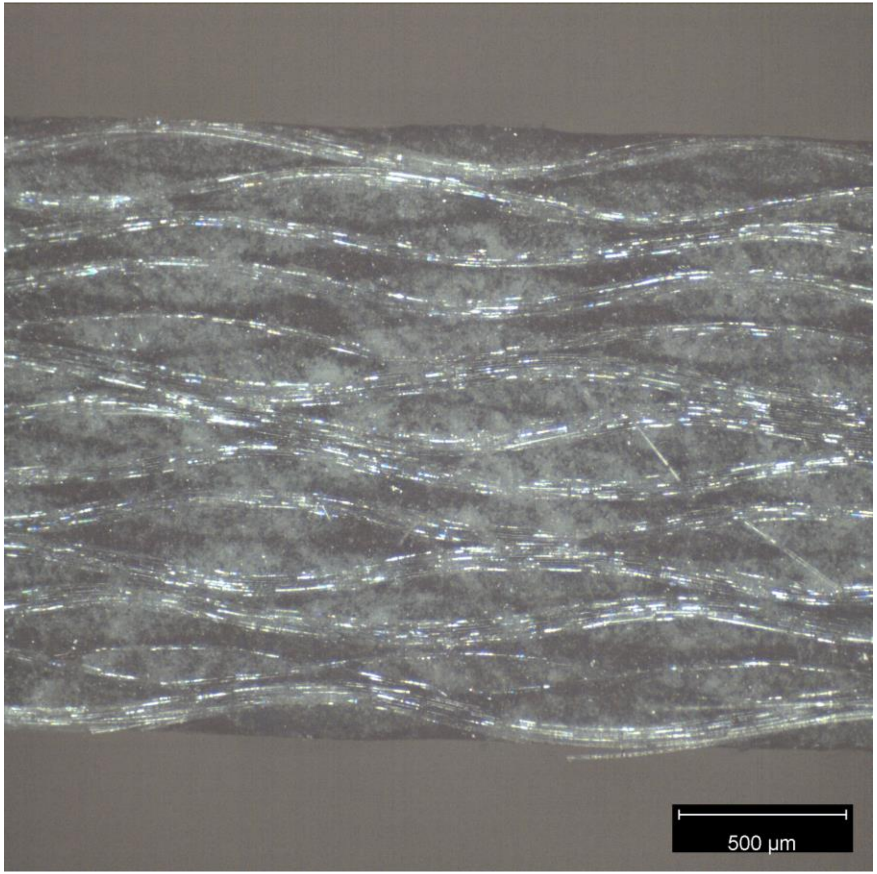	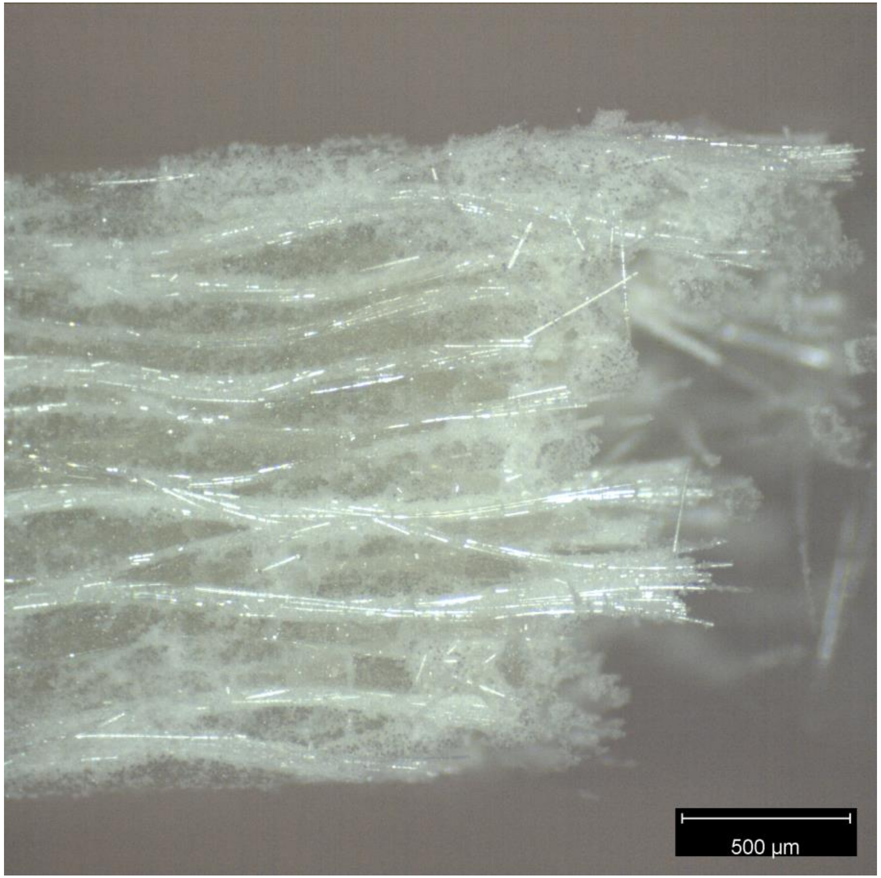

**EP_2_1**	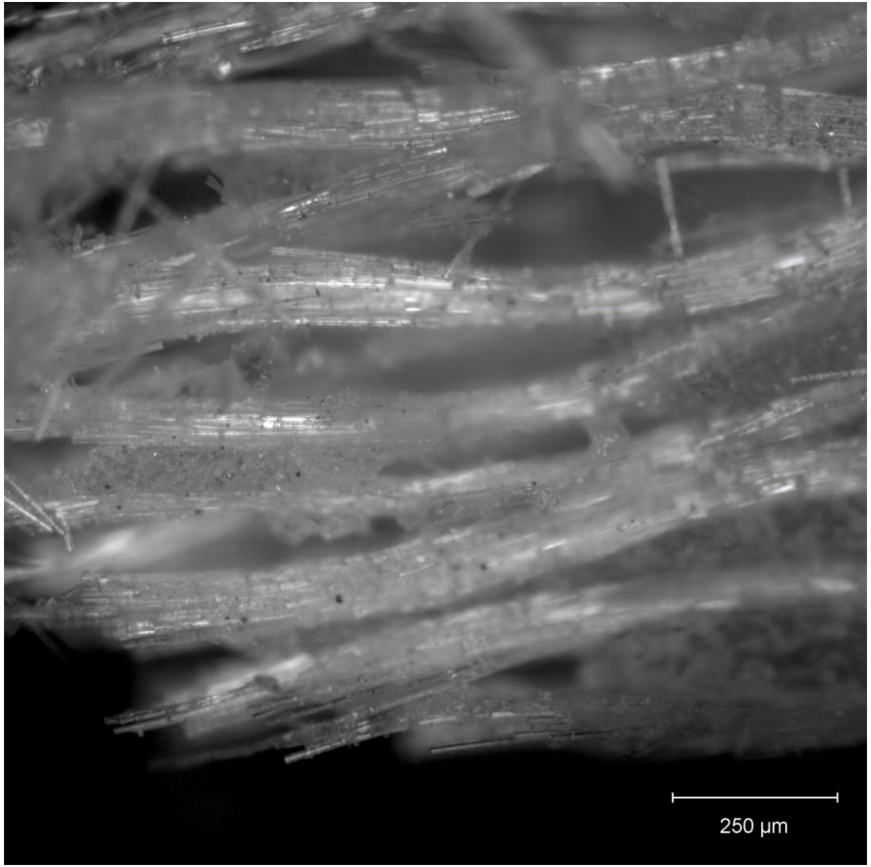	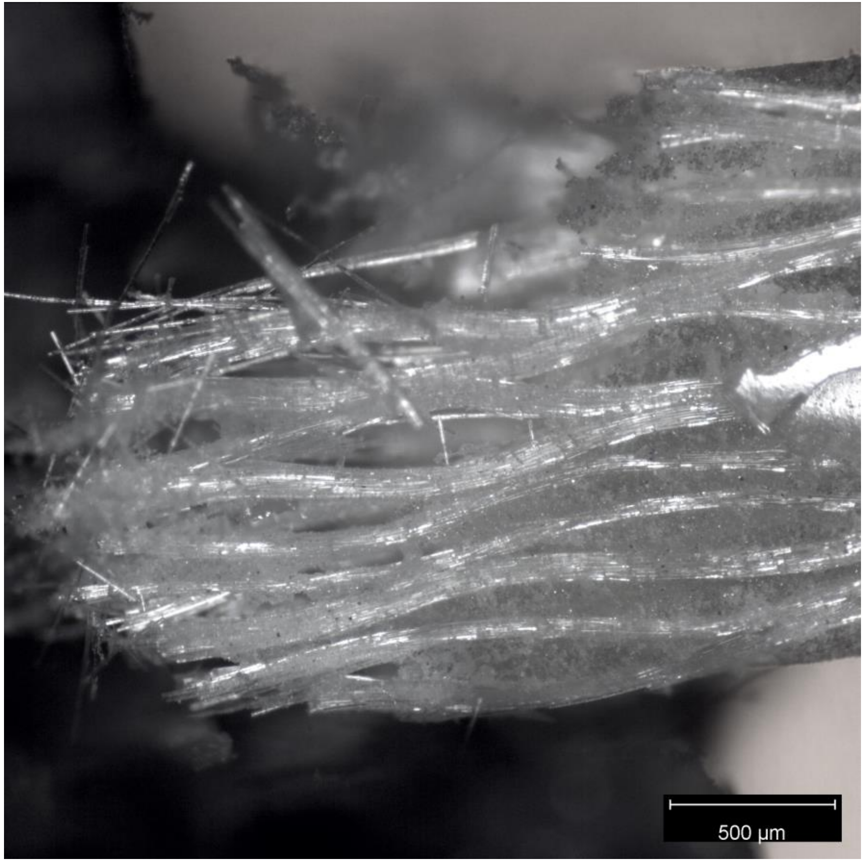

**EP_2_2**	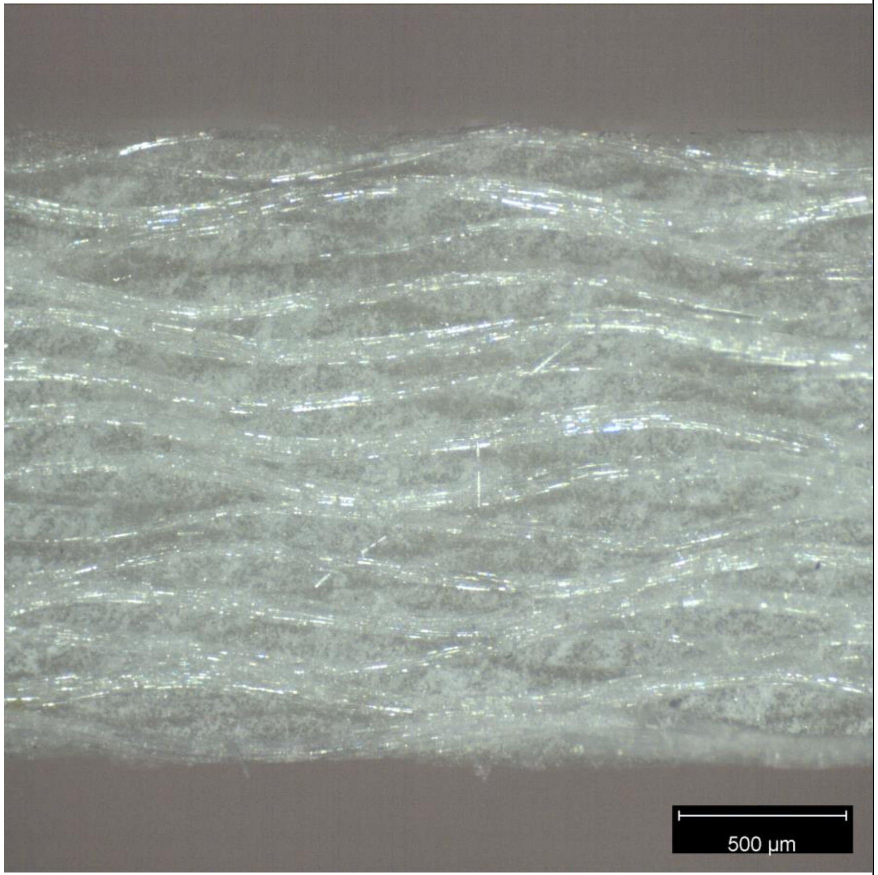	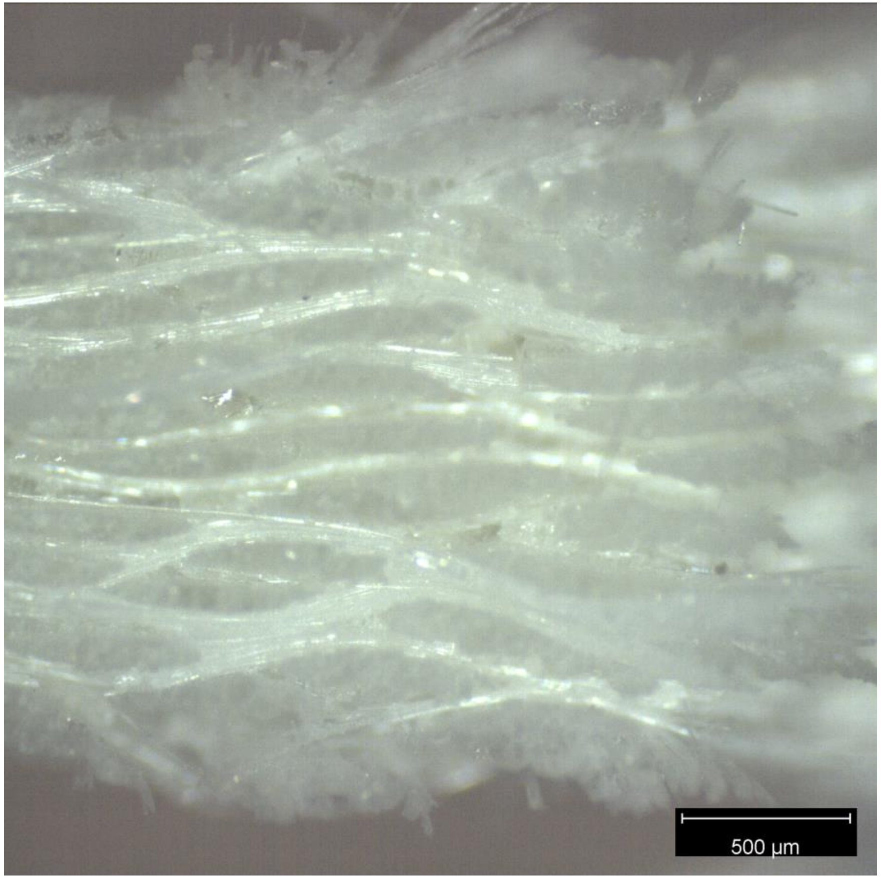

**EP_2_2**	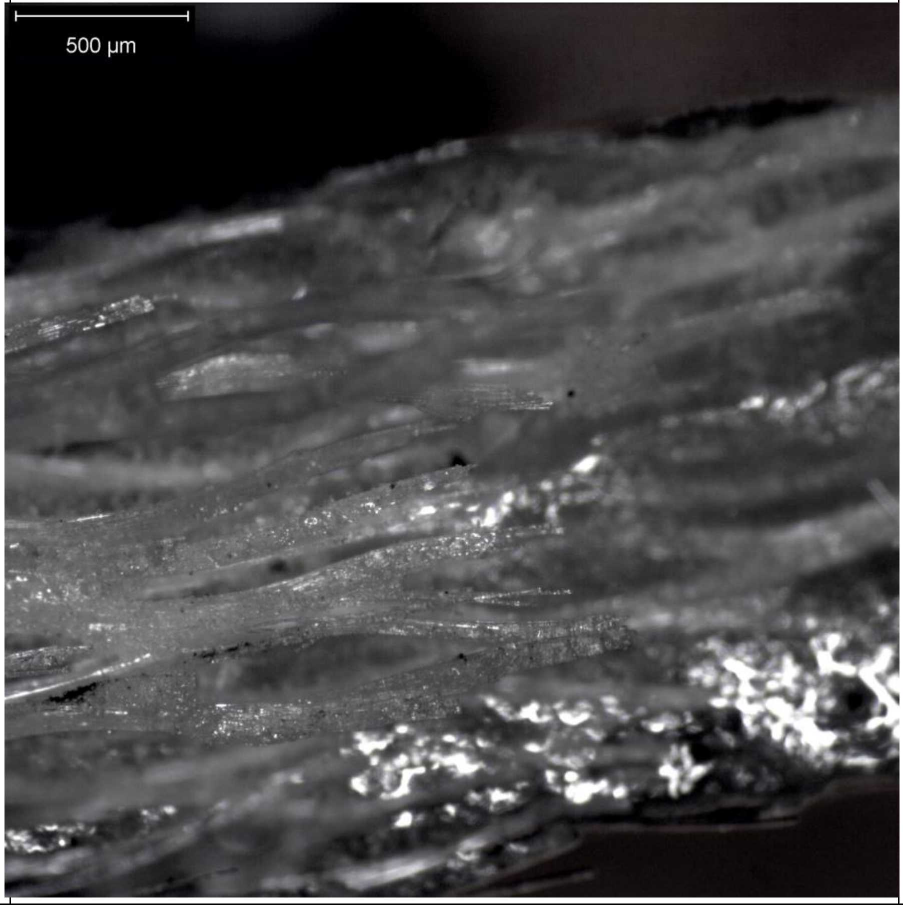	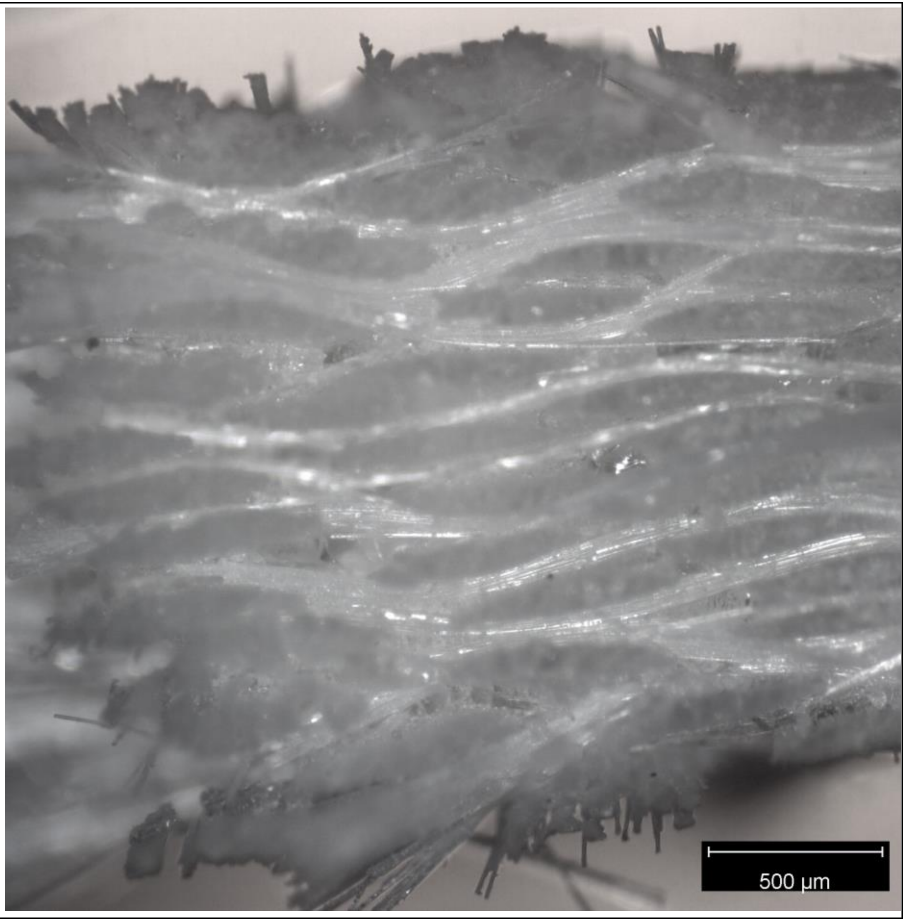

**EP_4_2**	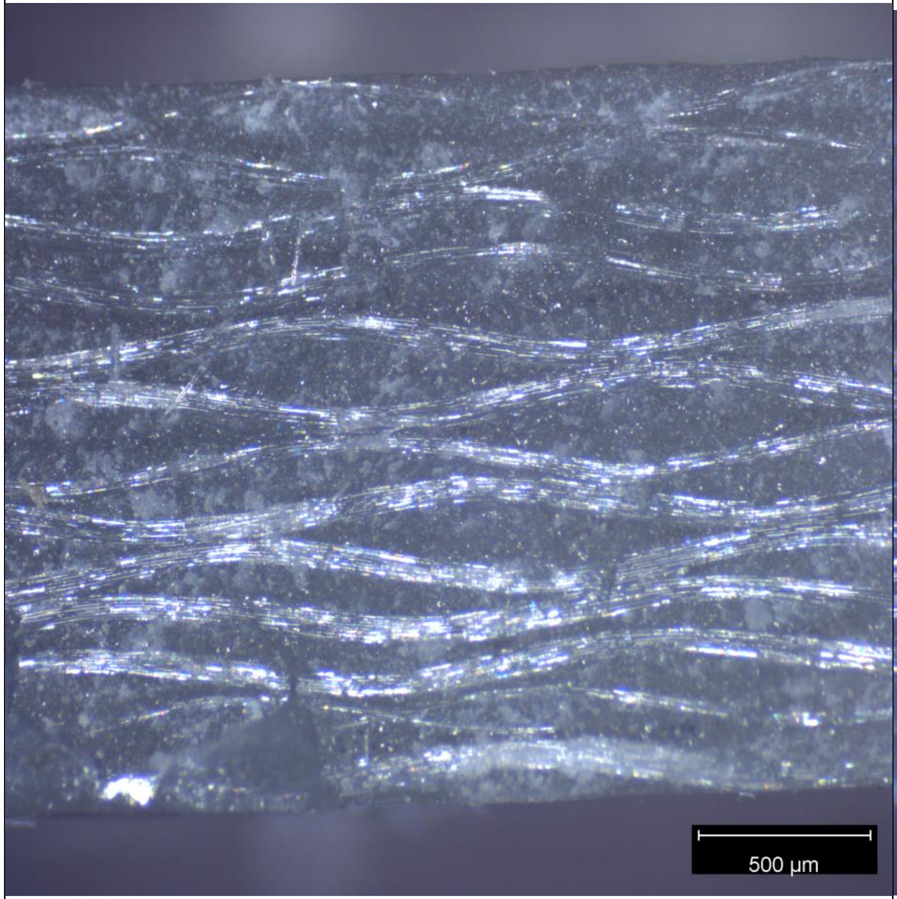	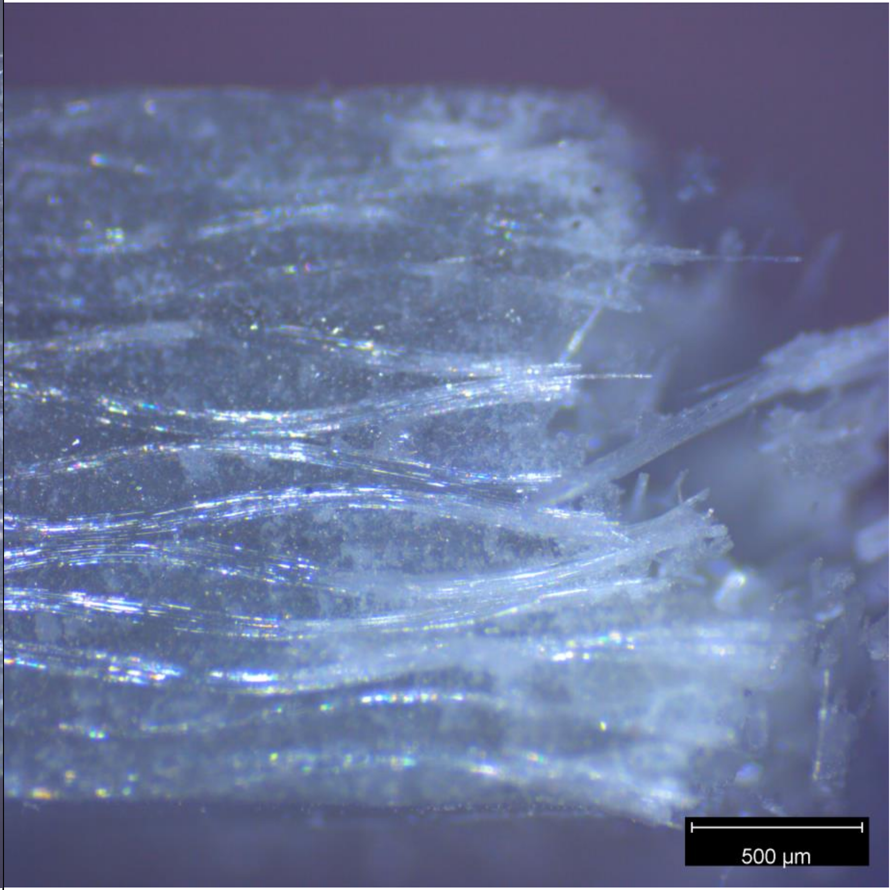

**EP_4_2**	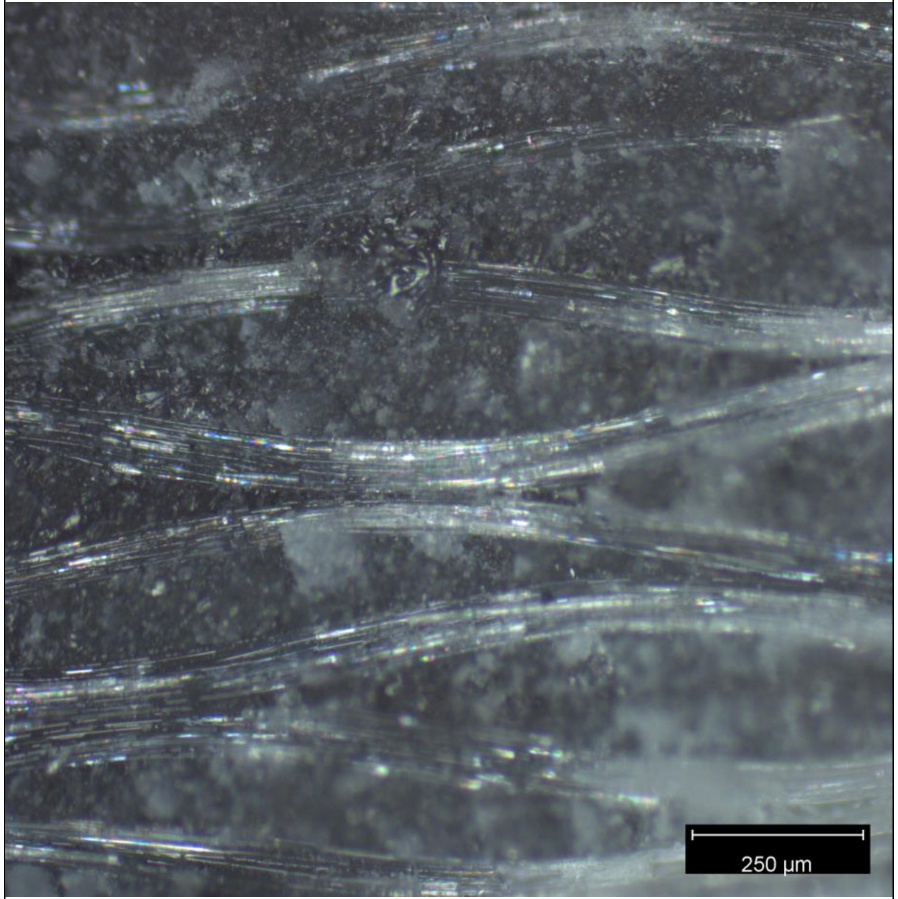	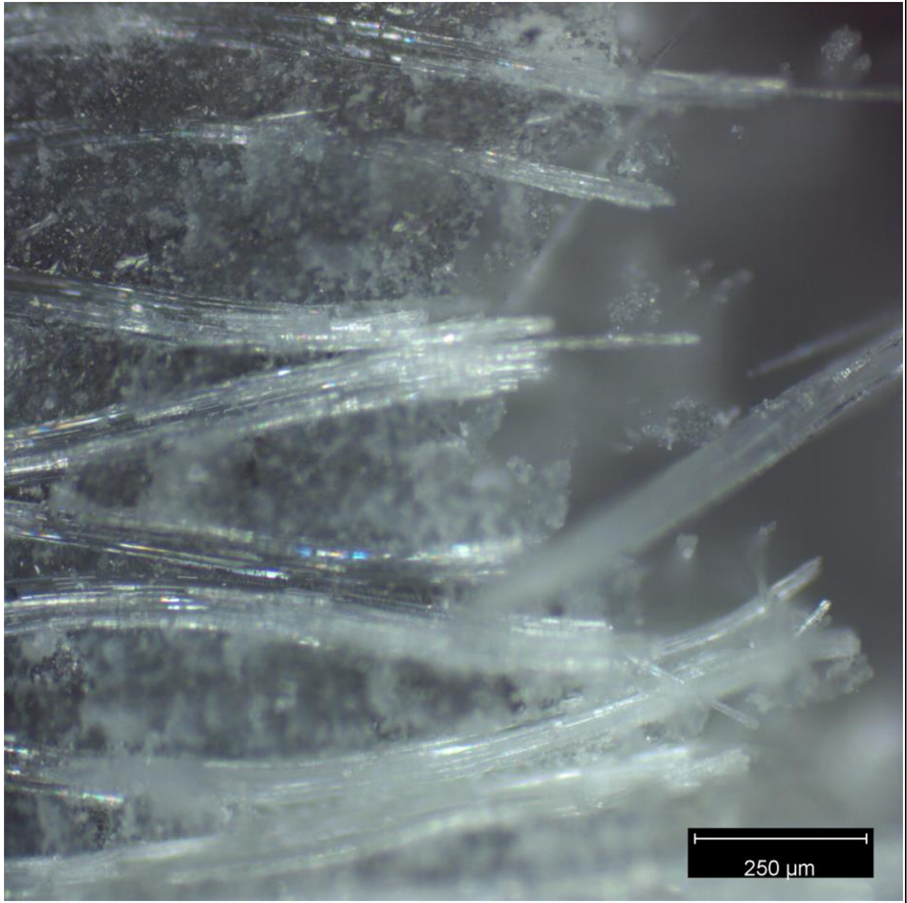
